# Fabrication of Transparent PEGylated Antifouling Coatings via One-Step Pyrogallol Deposition

**DOI:** 10.3390/polym15122731

**Published:** 2023-06-19

**Authors:** Shang-Lin Yeh, Piyush Deval, Wei-Bor Tsai

**Affiliations:** Department of Chemical Engineering, National Taiwan University, No. 1, Sec. 4, Roosevelt Rd., Taipei 10617, Taiwan; spy5162@psu.edu (S.-L.Y.); devalp.iitism@gmail.com (P.D.)

**Keywords:** polyethylene glycol, pyrogallol, antifouling

## Abstract

Antifouling coatings are critical for many biomedical devices. A simple and universal technique used to anchor antifouling polymers is important in order to expand its applications. In this study, we introduced the pyrogallol (PG)-assisted immobilization of poly(ethylene glycol) (PEG) to deposit a thin antifouling layer on biomaterials. Briefly, biomaterials were soaked in a PG/PEG solution and PEG was immobilized onto the biomaterial surfaces via PG polymerization and deposition. The kinetics of PG/PEG deposition started with the deposition of PG on the substrates, followed by the addition of a PEG-rich adlayer. However, prolonged coating added a top-most PG-rich layer, which deteriorated the antifouling efficacy. By controlling the amounts of PG and PEG and the coating time, the PG/PEG coating was able to reduce more than 99% of the adhesion of L929 cells and the adsorption of fibrinogen. The ultrathin (tens of nanometers) and smooth PG/PEG coating was easily deposited onto a wide variety of biomaterials, and the deposition was robust enough to survive harsh sterilization conditions. Furthermore, the coating was highly transparent and allowed most of the UV and Vis light to pass through. The technique has great potential to be applied to biomedical devices that need a transparent antifouling coating, such as intraocular lenses and biosensors.

## 1. Introduction

Biofouling, the accumulation of biomolecules or living microorganisms on surfaces of biomedical devices, can lead to detrimental clinical complications or impair the efficacy and performance of medical devices, such as blood-contacting devices, catheters, intraocular lenses, and biosensors [1,2,3,4]. For example, the nonspecific accumulation of plasma proteins on blood contact devices elicits platelet adhesion and activation, and subsequent thrombosis and biofilm formation [5,6,7]. The implantation of intraocular lenses for cataract correction induces a foreign body reaction to the implants and an epithelial cell reaction. To address these problems, antifouling coatings are essential for dealing with any biointerfacial materials that are exposed to biological fluids or tissues. A common antifouling strategy is the decoration of biomedical devices with hydrophilic polymers (such as poly(ethylene glycol) (PEG), poly-2-hydroxyethyl methacrylate, poly(2-hydroxypropyl acrylamide), dextran, and zwitterionic polymers) [8,9,10,11,12,13,14,15]. Among these antifouling polymers, PEG has been considered the “gold standard” in the pharmaceutical and biomedical industry. Thus, the surface immobilization of PEG is one of the most common approaches for fabricating antifouling surfaces.

The immobilization of PEG on biomaterial surfaces could be achieved via the physical adsorption or chemical conjugation of short-chain oligo(ethylene glycol) or long-chain PEG. The antifouling ability of surface-bound PEG is attributed to the excluded volume effect and the dynamic motion of hydrophilic PEO chains around a surface, and is dependent on the grafting density, chain length, and chain configuration [16,17]. Compared to physical adsorption, chemical conjugation is more robust and long-lasting. A common strategy to place a PEGylated adlayer on biomaterials involves the surface-induced polymerization of ethylene glycol methacrylate via surface-initiated atom transfer radical polymerization [18], plasma-induced graft polymerization [19,20], or radiation grafting [21]. However, these techniques could not be accessed by every laboratory or manufacturer due to the lack of indispensable equipment and specialty. Therefore, a facile and versatile method for the immobilization of PEG is welcomed in this field.

Mussel-inspired chemistry has been developed into a facile and universal technique for applications in diverse fields such as biomedical engineering, energy storage, and environmental issues [22]. The spontaneous self-polymerization of dopamine in an alkaline buffer deposits a thin, adherent, and robust polydopamine film on a wide variety of materials. Furthermore, polydopamine coatings can form chemical bonds with amino or thiol molecules, which is assumed via a nucleophilic reaction. By applying the polydopamine coating technique, our laboratory developed a simple one-step technique to immobilize antifouling polymers on various substrates [23,24,25]. Many plant-derived catecholic or galloyl compounds, such as tannic acid and pyrogallol, have recently attracted a great deal of attention due to the similarity in structure and surface deposition mechanism to dopamine [26]. Because these polyphenol compounds are extracted mostly from plants, they are much cheaper than dopamine. The oxidation of catecholic or galloyllic compounds initiates self-oligomerization into macrocompounds with higher molecular weights [27]. The poor solubility of macrocompounds, along with the inherent affinity for various materials, ultimately leads to surface deposition [28]. Catechol- and galloyl-based coatings possess a faster adhesion rate, excellent availability, and structural diversity than dopamine [29]. Furthermore, the characteristic dark brown color of the polydopamine coating impedes its application to some devices that require a highly transparent coating, in contrast to light brown polycatecholic or polygalloyl coatings [28], which broadens its potential applications.

The aim of this study was to fabricate a transparent antifouling coating via a pyrogallol (PG) deposition, which could be applied to devices that need transparency, such as intraocular lenses and inspection devices. We immobilized aldehyde-ended PEG onto several types of biomaterial substrates by simply immersing them in a PG/PEG solution. The antifouling performance of coatings was evaluated via their resistance to cell adhesion and protein adsorption. The correlation between antifouling efficacy and coating properties was evaluated. The stability and light transmittance properties of the coating were also investigated.

## 2. Materials and Methods

### 2.1. Materials

Chemicals: Pyrogallol (PG, cat#P0381), O-[2-(6-Oxocaproylamino)ethyl]-O′-methylpolyethylene glycol (aldehyde-ended PEG, cat#41964, molecular weight: 5000), O-(2-aminoethyl)polyethylene glycol (amine-ended PEG, cat# 672130, molecular weight: 5000), and dopamine hydrochloride (DOPA, cat#H8502) were purchased from Sigma-Aldrich (St. Louis, MO, USA). Gentamycin, 10X trypsin-EDTA, fungizone, and minimum essential medium alpha medium (α-MEM) were purchased from GIBCO (Grand Island, NY, USA). Fetal bovine serum (FBS) was purchased from JRH Biosciences (Brooklyn, VIC, Australia). Cell culture medium contained α-MEM supplemented with 10% FBS, 10 mL of fungizone, 5 mL of gentamycin, and 0.4 mL of 2-mercaptoethanol. Alexa Fluor 488-labeled fibrinogen (AF-Fib) was purchased from Thermo-Fisher Scientific (Waltham, MA, USA).

Substrates: Tissue cell polystyrene (TCPS) was obtained from Nunc (Rochester, NY, USA). Polydimethylsiloxane (PDMS; Sylgard 184) was obtained from Dow Corning (Auburn Hills, MI, USA). Polyethyleneterephthalate (PET), polystyrene (PS), polypropylene (PP), and poly(vinyl chloride) (PVC) were received from Nihon Shiyaku Industries Ltd. (Kyoto, Japan). The silicon wafer (Si) was obtained from Yia Chuan International Co. (Taoyuan, Taiwan). Polyurethane (PU), poly (vinylidene fluoride) (PVDF), poly (methyl methacrylate) (PMMA), and polylactic acid (PLA) substrates were fabricated using solution casting, as described in the Supplementary Materials.

### 2.2. Immobilization of PEG Mediated by Pyrogallol Self-Polymerization Deposition

PG dissolved in phosphate-buffered saline (100 mM PBS, pH 6.0) or DOPA dissolved in 10 mM tris buffer (pH 8.5) was mixed with the same volume of PEG solution of different concentrations in PBS. The mixtures were added to cover several types of substrates and then incubated at 45 °C under constant agitation. After incubation, the substrates were rinsed with deionized water and then air-dried. The above process was termed ‘one-step’, while the ‘two-step’ process started with PG solution (6 mg/mL) coating for 10 or 24 h, followed by incubation with PEG solution for the same period of time.

### 2.3. Characterization of PG/PEG Deposition

PG/PEG coatings were analyzed using a Fourier transform infrared spectrometer (ATR-FTIR, Spectrum 100, Perkin Elmer, Waltham, MA, USA) over 16 scans in the 1000~4000 cm^−1^ region using an attenuated total reflectance mode. XPS analysis was performed using an AXIS Ultra DLD spectrometer with a monochromated Al K_α_ source at a power of 45 W (15 kV × 3 mA) at a take-off angle of 72.5°. Surface hydrophilicity was evaluated using static contact angle measurement (FTA125, First Ten Ångstroms, Newark, CA, USA) at room temperature in at least 10 spots of each sample with 5 μL of deionized water. The thickness of the PG/PEG coatings was determined using spectral reflectance (Model F20, Filmetrics, San Diego, CA, USA) at more than 10 positions on each sample.

### 2.4. Evaluation of Fibrinogen Adsorption on PG/PEG-Coated PDMS

Fibrinogen adsorption to PG/PEG modified PDMS from a fluorescent-labeled fibrinogen solution was evaluated using total internal reflection fluorescence. Briefly, 0.5 mL of AF-Fib solution in PBS (0.1 mg/mL) was incubated with each modified PDMS sample at room temperature for 3 h, followed by several rinses with PBS to remove unbound AF-Fib. Surface fluorescence was visualized and recorded using fluorescent microscopy (Leica DM6000B Upright/Olympus IX71 Inverted Microscope System, Tokyo, Japan). For each sample, at least 10 images were randomly captured on each sample and the fluorescence intensities were analyzed using NIH ImageJ 1.53r software. A standard curve for the quantitation of fibrinogen adsorption was generated from a series of AF-Fib solutions with known concentrations.

### 2.5. Cell Adhesion to PG/PEG-Coated Surfaces

L929 cells were seeded on the PG/PEG modified substrates at a density of 2 × 10^4^ cells/cm^2^. After 24 h, the unattached cells were rinsed with PBS and the attached cells were imaged from optical microscopy. Five images were taken randomly from each sample (3 samples per substrate).

### 2.6. Characterization of PG/PEG-Coated PMMA

The light transmittance of modified PMMA was determined using UV-vis measurements (CARRY 300nc, Agilent Technology Santa Clara, CA, USA) at wavelengths ranging from 190 nm to 800 nm. The surface morphology of coatings on PMMA was analyzed using scanning electron microscopy (SEM, JSM530, JEOL, Tokyo, Japan). To evaluate the stability of the PG/PEG coating on PMMA, the substrates were autoclaved for 1 h at 122 °C and 1.2 kg/cm^2^, followed by cell culture to test the resistance to cell adhesion.

### 2.7. Statistical Analysis

The data were reported as means ± standard deviation (SD). Statistical analyses between different groups were determined using Student’s *t* test. The probabilities of *p* ≤ 0.05 were considered a significant difference. All statistical analyses were performed using the GraphPad Instat 3.0 program (GraphPad Software, La Jolla, CA, USA).

## 3. Results and Discussion

### 3.1. PG and PG/PEG Deposition

In our previous study, we demonstrated that PG deposition supported the immobilization of amine-containing zwitterionic polymers on several biomaterials, making them resistant to protein and cell attachment [9]. In that study, a copolymer of sulfobetaine methacrylate and 2-aminoethyl methacrylate was synthesized and deposited on various materials by dip-coating them with PG. While the sulfobetaine moiety provides antifouling ability, aminoethyl provides amino groups for conjugation with PG to anchor on substrates. However, this kind of approach requires the synthesis of a copolymer. It is convenient to use commercially available antifouling polymers for PG deposition. As PEG is the most widely used antifouling polymer, we can easily find PEG with a specific functional end, such as an amine or an aldehyde. In light of a study on prebiotic chemistry-inspired aminomalononitrile (AMN) polymerization [30], which creates surfaces similar to polydopamine, the authors showed that molecules that ended with an amine or an aldehyde could be conjugated to the AMN coating, so we also tested aldehyde-ended PEG for conjugation with PG in addition to amine-ended PEG in this study. Compared to the resistance to cell adhesion between both types of PEGs, we found that the deposition of aldehyde-ended PEG provided a better cell-resistant capacity; thus, in the subsequent experiment, aldehyde-ended PEG was used to fabricate the antifouling surfaces.

**Figure 1:**
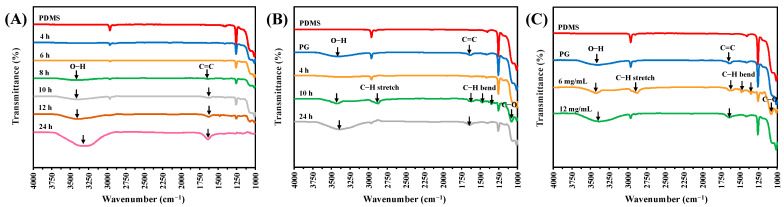
ATR-FTIR spectra for modified PDMS. (**A**) PDMS was coated with 6 mg/mL of PG at different times (4~24 h). (**B**) PDMS was coated with 6 mg/mL of PG and 20 mg/mL of PEG with different coating times (4, 10, and 24 h). (**C**) PDMS was coated with 6 mg/mL of PG with different amounts of PEG (4, 20, and 60 mg/mL) for 10 h.

The development of PEG/PG deposition on PDMS was first studied with respect to coating time (4, 6, 8, 10, 12, and 24 h) using ATR-FTIR. Characteristic PG peaks (1644 cm^−1^, attributed to the C=C stretching vibration of the aromatic ring, and 3424 cm^−1^, attributed to the O-H stretching vibration) were not detectable until 8 h after the deposition of 6 mg/mL of PG (Figure 1A), and became more apparent with increased coating time. The addition of 20 mg/mL of PEG gave characteristic PEG peaks after 4 h of coating, which became significant after 10 h (Figure 1B). However, after 24 h of coating, th characteristic PEG peaks decreased, while the characteristic PG peaks became prominent. We speculated that during deposition, PEG could be depleted after 10 h of coating, and then mainly polymerized PG was covered on the top layer. Therefore, the PEG signals could not be observed after 24 h.

Next, PEG was deposited at various concentrations (4, 20, or 60 mg/mL, along with a PG concentration of 6 mg/mL). PEG at 4 mg/mL could not provide sufficient PEG deposition for the appearance of characteristic peaks of PEG (Figure 1C). On the other hand, 60 mg/mL of PEG did not generate obvious characteristic PEG peaks either. We inferred that a large amount of PEG may interfere with PG in forming substantial aggregates to deposit on PDMS. From the above observations, an optimal ratio of PG/PEG and an optimal coating time were found to display a large amount of PEG on a substrate.

### 3.2. Cell Adhesion on PG/PEG Coatings

**Figure 2:**
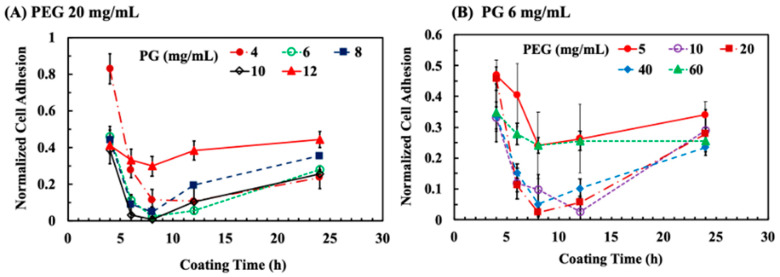
The adhesion of L929 cells on PG/PEG-coated PDMS. (**A**) Normalized cell adhesion to PDMS deposited with PEG 20 mg/mL and different PG concentrations for different coating times. (**B**) Normalized cell adhesion to PDMS deposited with PG 6 mg/mL and different PEG concentrations for different coating times.

The adhesion of L929 cells was carried out on a series of PG/PEG coatings on PDMS with different coating times (4, 6, 8, 12, and 24 h), PG concentrations (4, 6, 8, 10, and 12 mg/mL), and PEG concentrations (5, 10, 20, 40, and 60 mg/mL) in order to find optimal coating conditions that resist cell adhesion. PEG at a fixed concentration of 20 mg/mL was deposited with the PG concentration that ranged from 4 to 12 mg/mL. Cell adhesion was reduced to ~40% of original cell adhesion after 4 h of coating with most PG concentrations, except 4 mg/mL (Figure 2A). However, cell adhesion was reduced to ~28% on the PG/PEG coating by 4 mg/mL of PG after 6 h and further reduced to ~10% after 10 h. An increase in PG concentrations (6, 8, and 10 mg/mL) shortened the time to 6 h to reduce cell adhesion to 10%. The PG/PEG coating with 10 mg/mL for 8 h blocked 99.2% cell adhesion. However, cell adhesion on the PG/PEG deposited films bounced up after 8 h coating. After 24 h of deposition, cell adhesion increased to 20% (4, 6, and 10 mg/mL) or more (8 and 12%). Furthermore, with the deposition of PEG with 12 mg/mL of PG, 12 mg/mL only reduced cell adhesion to ~30% for 8 h, which returned to ~40% afterwards. We speculate that a large amount of PG causes the PG/PEG coating to be enriched with PG without sufficient PEG to resist cell adhesion.

Next, PEG ranging from 5 to 60 mg/mL was deposited at a fixed PG concentration at 6 mg/mL. After 4 h of coating, cell adhesion was reduced with PEG at all concentrations (Figure 2B). The deposition of 5 and 60 mg/mL of PG/PEG reduced cell adhesion to ~24% after 8 h of coating, which was not further reduced for a longer incubation time. Cell adhesion was reduced to a minimum of ~2.5% to the PG/PEG coating from 20 mg/mL with 8 h of incubation, and from 10 mg/mL but with longer incubation for 12 h. Similarly to the previous results, the inhibition of cell adhesion was reduced with a longer coating time. These results suggest that cell adhesion could be completely blocked on a PG/PEG coating with suitable amounts of PG (6–10 mg/mL) and PEG (10–20 mg/mL), with an incubation time of 6–12 h.

The deposition of PEG in this study was performed using a one-step coating approach, that is, from a mixture of PG and PEG. Some studies used a two-step coating approach to immobilize PEG, that is, the first deposition of PG, followed by the subsequent deposition of PEG [28]. For comparison between both methods, PEG was deposited using a one-step coating (PG/PEG = 6/20 mg/mL for 10 h) or a two-step coating (6 mg/mL of PG for 10 h followed by 20 mg/mL of PEG for 10 or 24 h). We found that cell adhesion was nearly completely blocked (~0.8%) to the substrate modified by the one-step coating method, while the inhibitory efficacy of the substrate modified by the one-step coating method was not as good even with 24 h of incubation (~8.0%) (Figure S1 in the Supplementary Materials). The result, similar to that of one study on polydopamine coating [23], suggests the advantages of the one-step coating method, that is, high efficacy with a short deposition time. We suggest that the PG layer may not provide sufficient binding sites for PEG conjugation in the two-step method.

### 3.3. PG/PEG Coatings to Various Substrate

**Figure 3:**
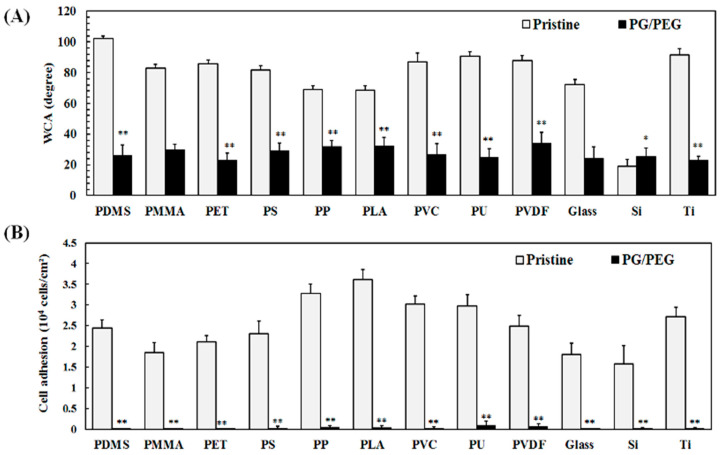
PG/PEG (6/20 mg/mL) was coated on a variety of materials for 10 h. (**A**) Static water contact angle measurement and (**B**) cell attachment. Value = mean ± standard deviation, *n* = 3. * indicates *p* < 0.01 and ** indicates *p* < 0.001 between the pristine and modified substrates: PDMS, polydimethylsiloxane; PET, polyethyleneterephthalate; PS, polystyrene; PP, polypropylene; PVC, poly(vinyl chloride); PU, polyurethane; PVDF, poly(vinylidene fluoride); PMMA, poly(methyl methacrylate); PLA, polylactic acid; Si, silicon wafer; Ti, titanium.

Polypyrogallol has affinity for a wide variety of substrates, such as metal, ceramic, and polymer substrates, as demonstrated in our previous work [31]. Therefore, PG/PEG should also be able to deposit on a variety of materials. In this study, a wide variety of commonly used biomaterials, such as PDMS, PET, PMMA, PP, PLA, PVC, PS, PU, PVDF, silicon (Si), glass, and titanium (Ti), were deposited by PG/PEG coatings in order to evaluate the antifouling efficacy. The PG/PEG coating effectively reduced the static water contact angles of all the substrates to around 30° even though the water contact angles of the pristine substrates varied (Figure 3A). Cell adhesion was almost totally inhibited by all PG/PEG modified materials (Figure 3B). The results demonstrated the universality of PG/PEG deposition to resist cell adhesion.

### 3.4. Surface Characterization of PG/PEG Coatings

**Figure 4:**
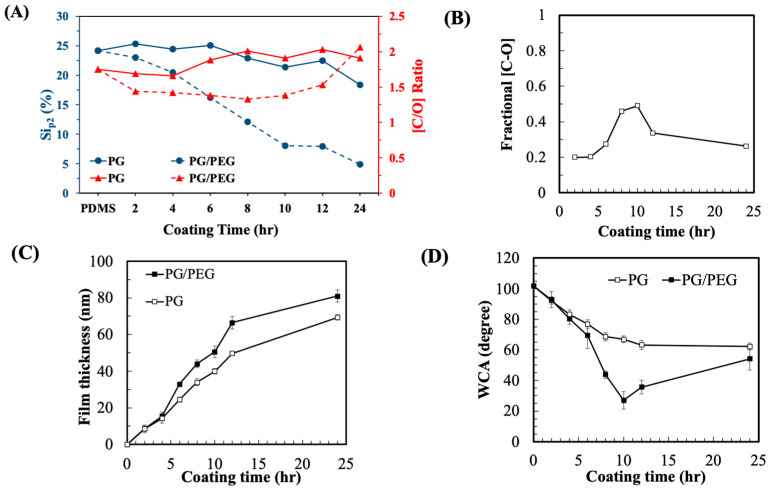
Characterization of PDMS deposited with PG (6 mg/mL) or PG/PEG (6/20 mg/mL) for different coating times (2~24 h). (**A**) XPS analysis of Si_p2_ peaks and the ratios of C/O peaks. (**B**) Fractions of [C-O] calculated from XPS C_1s_ high-resolution spectra of PDMS coated with PG/PEG. (**C**) The thickness of the film deposited to the silicon wafer determined using spectral reflectance, *n* > 10. (**D**) Static water contact measurement, *n* > 10.

To understand the mechanism of PG/PEG deposition, PDMS was deposited with a series of PG/PEG coatings with different incubation times ranging from 2 to 24 h. These modified surfaces were characterized using ESCA analysis. PG gradually deposited on the surfaces of PDMS with time, indicated by the fact that the content of the Si element of PDMS, which does not exist in PG, decreased with time, from 24.2% of the pristine PDMS to 18.4% after 24 h of coating (Figure 4A). On the other hand, the co-deposition of PG/PEG decreased the Si signal much more rapidly from 24.2% of PDMS to 4.9% after 24 h of coating, indicating that the PG/PEG adlayer of PG/PEG grows faster than the PG adlayer. Although the C/O elemental ratios were close between the PDMS, PG, and PEG molecules, the C/O ratios calculated from the XPS data were quite different among the PDMS-, PG-, and PG/PEG-coated samples. PG deposition gradually increased the C/O ratio from 1.75 of PDMS to ~2 after 8 h of incubation, while PG/PEG deposition initially decreased the C/O ratio to 1.33 after 8 h of incubation and then increased to ~2.0 after 24 h of incubation. The XPS results of PG/PEG are consistent with the FTIR data (Figure 1), showing that PEG is initially deposited at the top of the coating and then covered by PG.

We also analyzed the high-resolution C_1s_ spectra (Figure S2 in supporting information). The shake-up peak for the aromatic carbon of pyrogallol was apparent after 10 h of PG incubation (Figure S2 in the Supplementary Materials). On the PG/PEG coating, the characteristic PEG [C-O] peak at ~286.7 eV appeared after 8 h of deposition, reached a maximum at 10 h, followed by shrinkage after 24 h (Figure S2). The fractional area of the [C-O] peak of PEG in the C_1s_ spectra can be taken as a measure of the density of the grafting of PEG [32]. The PEG content was detected after 4 h of coating and then increased to a maximum after 10 h of coating, followed by a decrease afterwards (Figure 4B).

The thickness of the PG and PG/PEG coatings increased with time (Figure 4C). During the initial 4 h, there was no significant difference in film thickness between the PG and PG/PEG deposition, while the PG/PEG film grew faster than the PG film during the next 8 h. However, the difference in film thickness between the two coating did not increase afterwards.

The deposition of PG and PG/PEG was also demonstrated by surface wettability in terms of water contact angles (WCAs). PG deposition decreased the WCA from 101.6° of pristine PDMS to 66.9° after 10 h of coating, and the WCA remained stable afterwards (Figure 4D). The deposition of PG/PEG exhibited a similar WCA (80.1°) to PG during the first 4 h of coating, but the WCA decreased significantly afterward and reached a minimum of 27° after 10 h of coating. The WCA was reversed for continuous coating to 35.6° and 54.0° at 12 and 24 h, respectively.

From the surface analysis data, the development of PG/PEG could be divided into three stages. During the first 4 h, the adlayer was mainly composed of PG with little PEG. During the second stage of the coating (4–10 h), the immobilization of PEG increased exponentially, indicated by the increase in film thickness and the decrease in water contact angles. In the final stage, PEG might be depleted in the PG/PEG solution, so the PEG-rich adlayer was covered by PG-rich deposition, which is demonstrated by the XPS and water contact angle data. The coating mechanism provides us with important information to optimize the conditions for the antifouling coating.

### 3.5. Correlation between Surface Characteristics and Antifouling Efficacy

**Figure 5:**
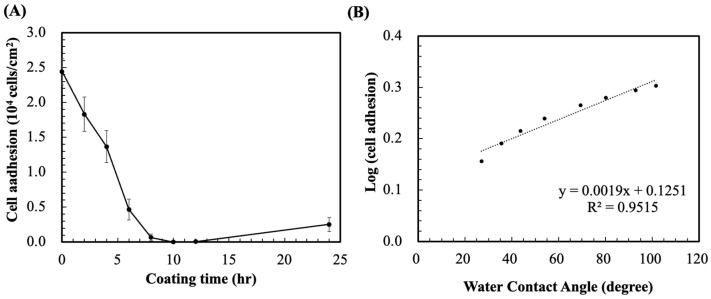
(**A**) L929 cell adhesion results for 24 h (scale bar = 100 μm) and (**B**) fibrinogen adsorption on PG/PEG (6/20 mg/mL) coated PDMS controlled for different coating times. Value = mean ± standard deviation, *n* = 3.

Next, we evaluated the antifouling efficacy of PG/PEG surfaces in terms of the adhesion of L929 fibroblasts. A significant decrease in cell adhesion was observed with an increase in coating time (Figure 5A). Cell adhesion was significantly blocked after merely 2 h of PG/PEG coating, from the initial 2.4 × 10^4^ cells/cm^2^ on pristine PDMS to 1.83 × 10^4^ cells/cm^2^. Resistance to cell adhesion was further enhanced with coating time, and cell adhesion was almost inhibited after 10 and 12 h of incubation (19 and 58 cells/cm^2^, respectively). Cell adhesion increased slightly afterward and was 2500 cells/cm^2^ after 24 h of coating.

The antifouling ability of a surface is related to its wettability, so we plotted the cell adhesion data against the surface wettability (Figure 5B). It was observed that the log of cell adhesion had a positive linear correlation with WCA. Therefore, one can easily predict the resistance of a surface to cell adhesion simply from its water contact angle.

**Figure 6:**
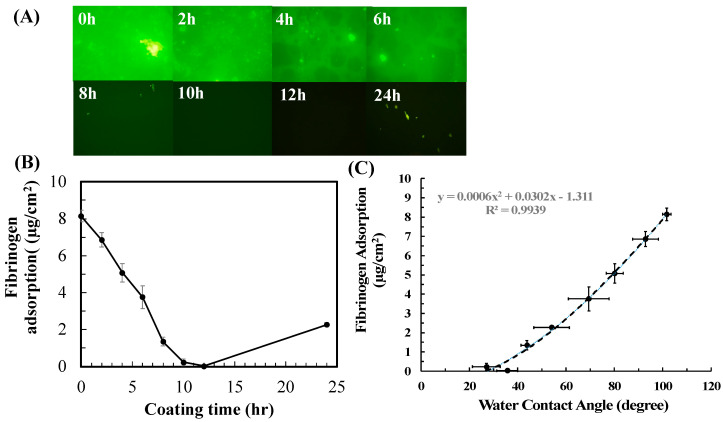
Adsorption of AF-Fib adsorption to PDMS coated with PG/PEG (6/20 mg) for different incubation times. (**A**) Fluorescent images of adsorbed AF-Fib at different time points. (**B**) Quantified fibrinogen adsorption. (**C**) Correlation between fibrinogen adsorption and water contact angles. Value = mean ± standard deviation, *n* = 10.

Cell adhesion to a biomaterial was mediated by protein adsorption [6], so protein adsorption to PDMS coated with PG/PEG was also measured. Fibrinogen, the major plasma protein that mediates platelet adhesion and activation [33], was used as a model protein in this study. Similarly to cell adhesion, fibrinogen adsorption decreased with coating time until 12 h after coating and then rebounded slightly afterward (Figure 6). Compared to pristine PDMS (8.143 µg/cm^2^), fibrinogen adsorption decreased to 6.853 µg/cm^2^ after 2 h of coating and then decreased further to 0.026 µg/cm^2^ for 12 h of coating. Due to PG deposition on the PEG coating afterward, fibrinogen adsorption increased to 2.262 µg/cm^2^ after 24 h of coating.

### 3.6. Deposition of PG/PEG to PMMA

**Figure 7:**
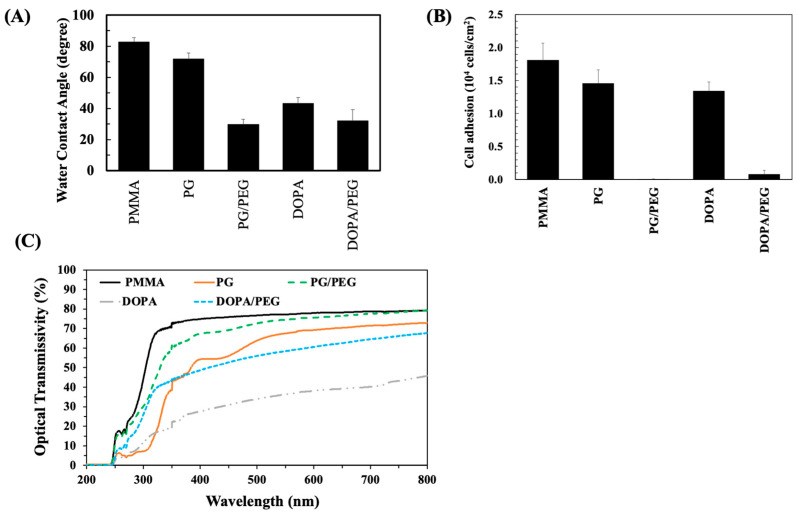
PMMA was coated with PG/PEG or DOPA/PEG (6/20 mg/mL) for 10 h. (**A**) Water contact angle results on coated PMMA. (**B**) L929 cell adhesion results on coated PMMA. (**C**) Optical transmissivity through the coatings.

A transparent antifouling coating is important for a wide variety of medical devices, such as intraocular lenses and inspection devices. We found that the PG/PEG coating is quite transparent, so the coating might benefit such applications. PMMA, one of the most widely used materials for intraocular lenses, was modified by PG/PEG deposition for 10 h and then surface properties were investigated. To compare the PG and DOPA coatings, PMMA was deposited with DOPA/PEG using the same concentrations as those for the PG/PEG coating. Pristine PMMA is a moderately hydrophobic material with a WCA of ~83° (Figure 7A). PG deposition slightly reduced WCA to 72°, while the dopamine coating was more hydrophilic (~43°). The incorporation of PEG drastically reduced WCA to ~30° for both PG/PEG and DOPA/PEG. Cell attachment to PMMA coated with PG and DOPA was close. The PG/PEG coating resisted almost 100% cell adhesion, while the DOPA/PEG coating resisted 8% cell adhesion (Figure 7B). The transmittance of UV-vis lights from 200 to 800 nm through modified PMMA was analyzed. Compared to pristine PMMA, while the PG and DOPA coatings blocked most UV light, the DOPA coating only allowed less than 40% of the transmittance of visible light, but the PG coating blocked less than 50% of visible light (Figure 7C). The addition of PEG improved the transmittance of both PG and DOPA coatings. The DOPA/PEG coating greatly enhanced the transmittance of the DOPA coating, especially for UV light. However, DOPA/PEG was inferior to PG in transmittance in the visible light region. On the contrary, the PG/PEG coating was clear enough to allow most of the transmittance of UV and Vis light to pass through. Here, we show that although the deposition of PEG via PG or DOPA is similar in terms of resisting cell adhesion, the PG/PEG coating is much more transparent than the DOPA coating.

**Figure 8:**
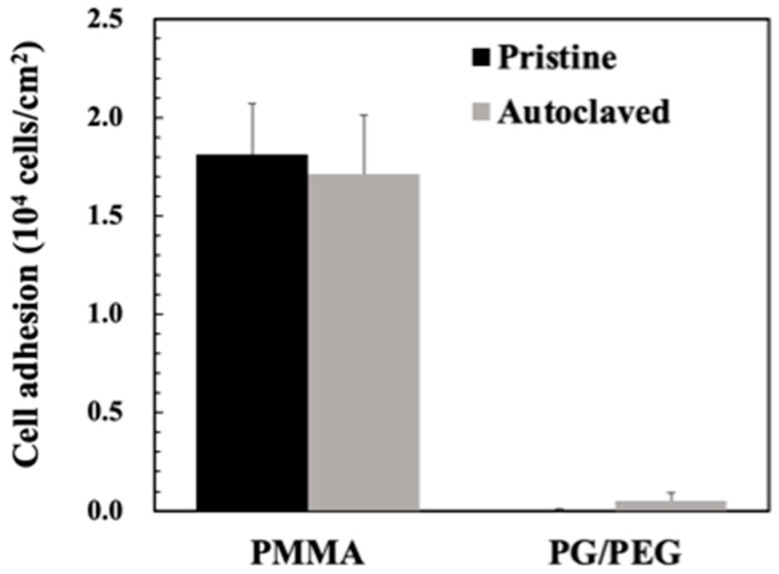
Cell adhesion to PMMA or PMMA coated with PG/PEG before and after autoclaving.

The morphology and stability of the PG/PEG coating were also studied. SEM images of modified PMMA showed that PG aggregates were occasionally found on the PG-coated surfaces, while the PG/PEG coating remained smooth morphology, similar to pristine PMMA (Figure S3 in Supporting Information). Another issue for surface coatings of biomedical devices is the requirement for sterilization prior to clinical usage, which may damage the coatings. PG/PEG-coated PMMA was subjected to autoclaving at high temperatures and pressure levels. We found that autoclaved PG/PEG-modified PMMA allowed slight cell adhesion (Figure 8), which could be due to the detachment of the coating or the degradation of PEG at high temperatures and pressure levels. However, our results suggest the robustness of the PG/PEG coating on PMMA. Overall, the transparent and robust PG/PEG coating could be easily deposited onto a wide variety of materials and effectively resist cell adhesion and protein adsorption. The technique has great potential to be applied to biomedical devices that need a transparent antifouling coating.

## 4. Conclusions

In this study, we developed a simple one-step technique for the PEGylation of biomaterial surfaces via the self-polymerization of pyrogallol. PEG containing an aldehyde end together with PG was firmly anchored onto various substrates and showed excellent resistance to cell adhesion and protein adsorption. Notably, the PG/PEG coating is highly clear and is compatible for devices that require transparency such as intraocular lenses and biosensors.

## Data Availability

Not applicable.

## Supplementary Materials

The following supporting information can be downloaded at: https://www.mdpi.com/article/10.3390/polym15122731/s1. Figure S1: Cell adhesion on PDMS that was modified using 1-step (1S) or 2-step (2S) coating; Figure S2: High resolution of C1s in XPS spectra for PDMS coated with PG/PEG for different times; Figure S3: SEM images of PMMA coated with PG or PG/PEG (6/20 mg/mL) for 10 h.
