# Supplementary material for: Fabrication of Transparent PEGylated Antifouling Coatings via One-Step Pyrogallol Deposition
Source: Polymers (Basel). 2023 Jun 19;15(12):2731. doi: 10.3390/polym15122731 (PMC10302030; doi:10.3390/polym15122731)
Supplement: Supplementary file 1 [file polymers-15-02731-s001.zip › polymers-2421637-supplementary.pdf]

Supporting information

1. Processes for fabrication of substrates
   (1) To prepare polyurethane (PU) substrates, waterborne polyurethane was dropped in glass dish with 5 cm in diameter and then dried overnight in a vacuum oven at 100°C.
   (2) To prepare Poly(vinylidene fluoride) (PVDF) substrates, PVDF powder was dissolved in 1-Methyl-2-pyrrolidinone (NMP) (5%, w/w). The mixture was spread on the glass slide as a film and then dried in a vacuum oven at 90°C to obtain the PVDF film.
   (3) To prepare Poly(methyl methacrylate) (PMMA) substrates, PMMA powder was dissolved in acetone solution (4%, w/v). The mixture was spread on the alumni plate and the solvent was evaporated on a 50℃ heating plate to obtain the PMMA film.
   (4) To prepare Polylactic Acid (PLA) substrates, PLA powder was dissolved in chloroform (5%, w/v). The mixture was spread on the glass plate and the solvent was evaporated on a 60℃ heating plate to obtain the PLA film.

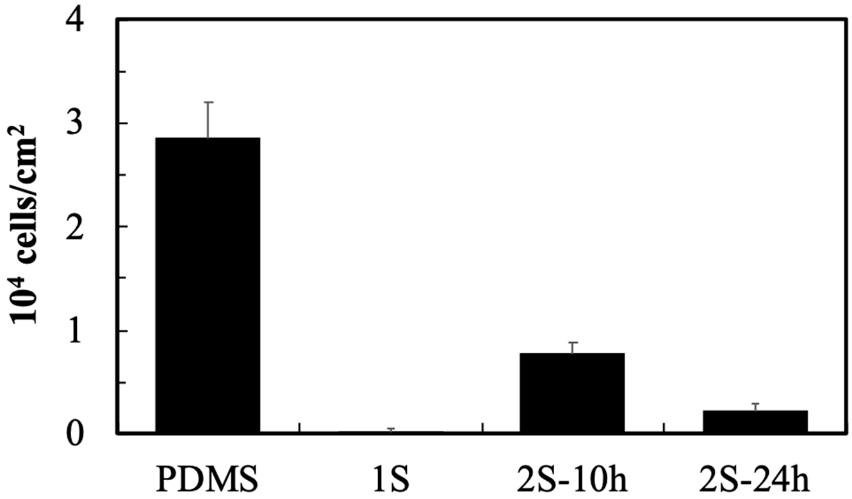

Figure S1. Cell adhesion on PDMS that was modified using 1-step (1S) or 2-step (2S) coating. For 1S coating, PDMS was deposited with 6 mg/mL PG and 20 mg/mL PEG for 10 hours; for 2S coating, PDMS was first deposited with 6 mg/mL PG solution for 10 h, followed by the deposition of 20 mg/mL PEG incubation for 10 (10h) or 24 hours (24h).

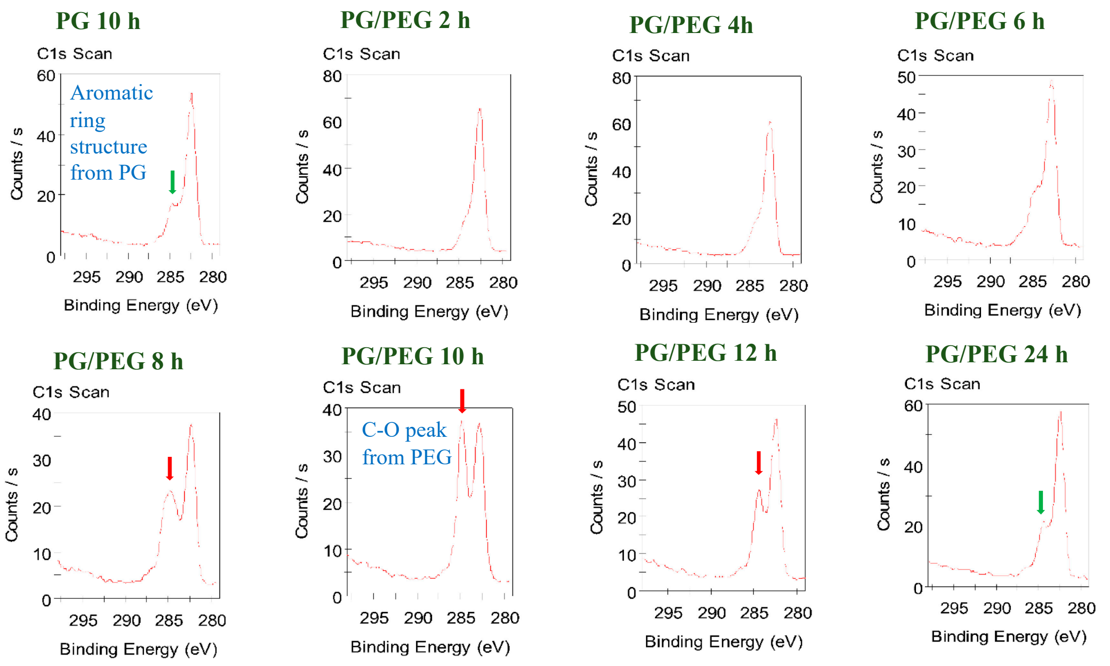

Figure S2. High resolution of C_{1s} in XPS spectra for PDMS coated with PG/PEG for different times. The shake-up peak for the aromatic carbon of pyrogallol and the peak for [C-O] for PEG are indicated by green and red arrows, respectively.

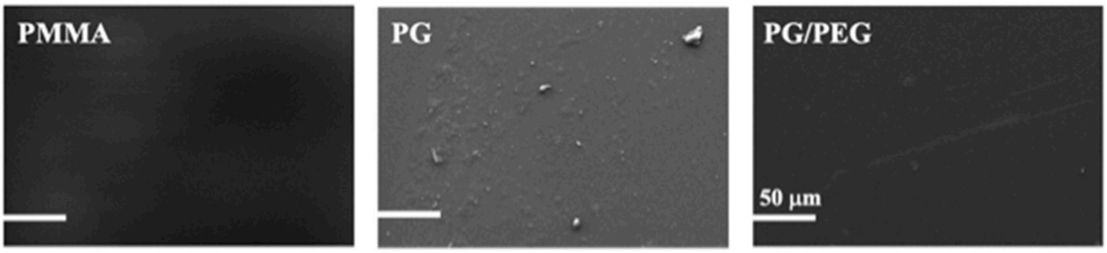

Figure S3. SEM images of PMMA coated with PG or PG/PEG (6 /20 mg/mL) for 10 hours.
